# Electromagnetic field exposure (50 Hz) impairs response to noxious heat in American cockroach

**DOI:** 10.1007/s00359-018-1264-2

**Published:** 2018-05-02

**Authors:** Justyna Maliszewska, Patrycja Marciniak, Hanna Kletkiewicz, Joanna Wyszkowska, Anna Nowakowska, Justyna Rogalska

**Affiliations:** 1 Department of Animal Physiology, Faculty of Biology and Environmental Protection, Nicolaus Copernicus University, ul. Lwowska 1, 87-100 Toruń, Poland; 2 Nicolaus Copernicus University, Toruń, Poland; 3 Department of Biophysics, Faculty of Biology and Environmental Protection, Nicolaus Copernicus University, Toruń, Poland

**Keywords:** American cockroach, Electromagnetic field, Glutathione, Heat nociception, Lipid peroxidation

## Abstract

Exposure to electromagnetic field (EMF) induces physiological changes in organism that are observed at different levels—from biochemical processes to behavior. In this study, we evaluated the effect of EMF exposure (50 Hz, 7 mT) on cockroach’s response to noxious heat, measured as the latency to escape from high ambient temperature. We also measured the levels of lipid peroxidation and glutathione content as markers of oxidative balance in cockroaches exposed to EMF. Our results showed that exposure to EMF for 24, 72 h and 7 days significantly increases the latency to escape from noxious heat. Malondialdehyde (MDA) levels increased significantly after 24-h EMF exposure and remained elevated up to 7 days of exposure. Glutathione levels significantly declined in cockroaches exposed to EMF for 7 days. These results demonstrate that EMF exposure is a considerable stress factor that affects oxidative state and heat perception in American cockroach.

## Introduction

Exposure to electromagnetic fields has become inescapable, especially at extremely low frequencies (30–300 Hz) given off by electrical appliances and overhead power lines. Therefore, more concerns are given about the potential adverse health effects of EMF exposure. It was shown that EMF can act as a stressor and may activate a wide spectrum of interacting neuronal, molecular and neurochemical systems that underpin behavioral and physiological responses (Levin 2003; Wyszkowska et al. 2006; Blank and Goodman 2009; Zeni et al. 2017). The effects of EMF exposure on insect morphology, physiology and behavior have been proved previously. The EMF exposure induced changes in: mosquito egg hatching (Pan and Liu 2004), ovipositon in *Drosophila* (Gonet et al. 2009), locomotor activity of desert locust and American cockroach (Wyszkowska et al. 2006, 2016) or antioxidant defense in *Baculum extradentatum* (Todorović et al. 2012). EMF exposure has been also shown to induce a release of octopamine—an insect ‘stress hormone’ in American cockroach (Wyszkowska et al. 2006), whereas the static electric field exposure elevated octopamine levels in *Drosophila* brain (Newland et al. 2015).

Exposure to EMF, similar to other stress factors, has been shown to trigger oxidative stress, observed as the increase of lipid and protein oxidative damage in various tissues. Moreover, significant changes in levels of antioxidants, such as glutathione, superoxide dismutase or catalase were observed (Kivrak et al. 2017). Zhang et al. (2016) demonstrated that thermal stress (35 °C) and EMF exposure (50 Hz, 3 mT) elicit a synergistic effect, strengthening the negative effect of EMF on lifespan, locomotion and oxidative stress in *Drosophila melanogaster*.

Strong stress reduces the sensitivity to pain. However, it has been demonstrated that in mice, acute exposure to electromagnetic field suppresses the stress-induced analgesia and works in a similar way to nalaxone, an antagonist of the opioid system (Kavaliers and Ossenkopp 1994). Insects’ nociceptors that respond to harmful stimuli, such as members of transient receptor potential (TRP) family, are the conserved molecular basis for the perception of noxious stimuli in vertebrates and invertebrates (Im and Galko 2012). It has been shown that nociceptive response is modified after EMF exposure. In rats exposed to 0.25 µT EMF analgesic response, equivalent to the effect of 4 mg/kg of morphine was observed (Martin et al. 2004). Moreover, in land snail *Capaea nemoralis*, EMF exposure (60 Hz, 100µT) attenuated the response to thermal nociceptive stimuli (Tysdale et al. 1991).

Thus, we put forward the hypothesis that electromagnetic field alters the response to noxious heat in insects. To test this hypothesis, the effect of EMF exposure (50 Hz, 7 mT) on cockroach’s response to noxious heat was examined. Moreover, we evaluated the level of oxidative stress in cockroaches exposed to EMF. The parameters of exposure used in our experiments are commonly applied in magnetotherapy (Karpowicz 2015).

## Materials and methods

### Animals

The experiments were performed on adult males of American cockroach *Periplaneta americana* L. Cockroaches were reared in plastic cages at constant temperature 26 ± 2 °C, with relative humidity 40% and 12:12 light–dark regime.

### Electromagnetic field exposure system

**Fig. 1:**
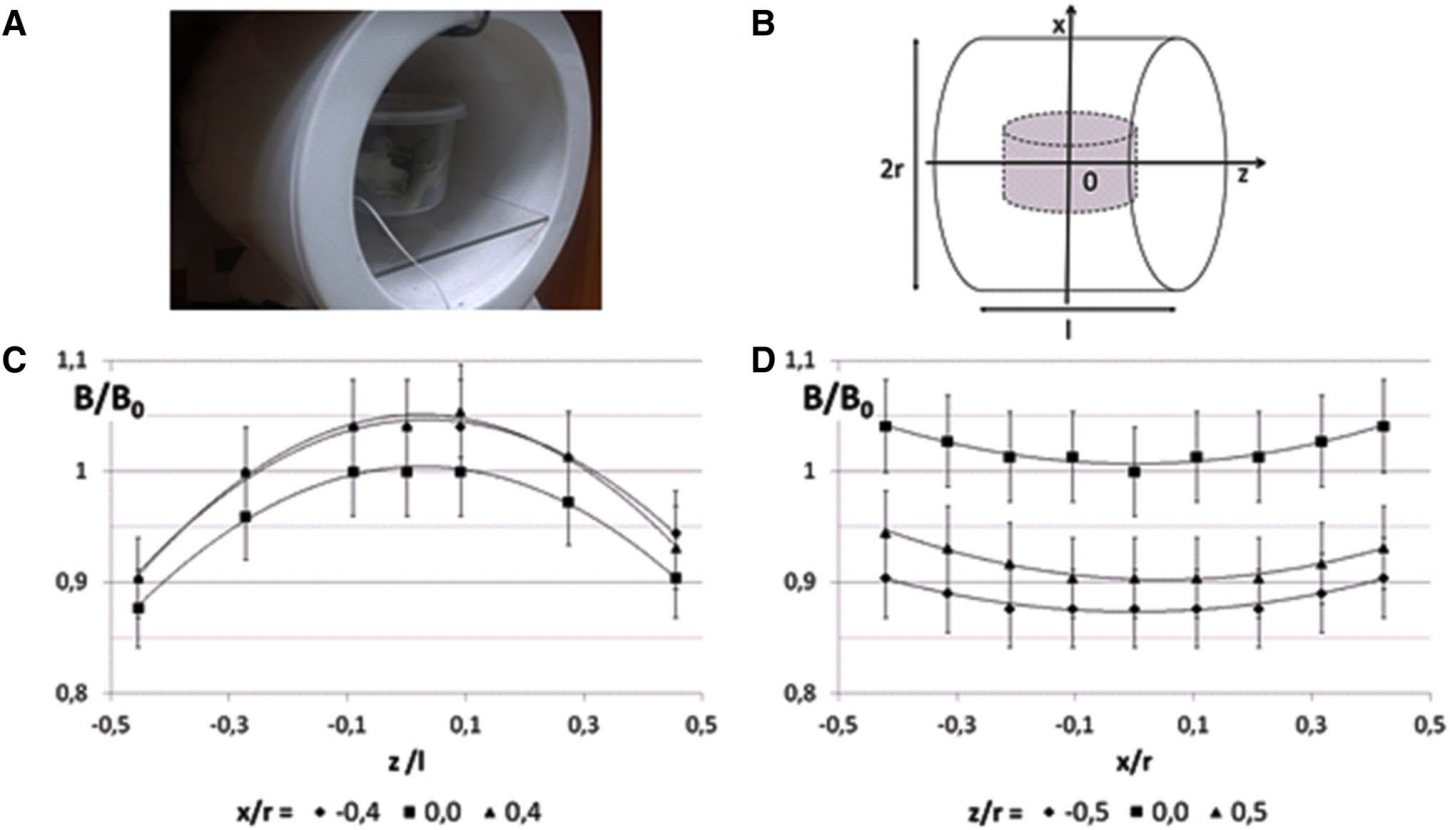
The exposure system. Cockroaches in the magnetic coil (**a**). The coordinate system (**b**). The magnetic flux density distribution inside the solenoid along *Z*-longer axis (**c**) and *X*-radial axis (**d**)

Electromagnetic field (EMF) with the domination of magnetic component was generated by a single 20 cm diameter coil (Elektronika i Elektromedycyna Sp. J.; Poland), as was previously described (Bieńkowski and Wyszkowska 2015) (Fig. 1a). The coil produced homogeneous, sine-wave alternating electromagnetic fields at 50 Hz with the intensity of 7 mT. The distribution of magnetic flux density within the coil along Z and X axes is shown on Fig. 1b–d. The maximum homogeneity inside the coil was 10%. The magnetic field level was controlled before each experiment using a Gaussmeter (Model GM2, AlphaLab, Inc, USA).

Animals were exposed to EMF inside the coil. The cockroaches (*n* = 20) were placed together in a cylindrical glass chamber (10 cm × 7.5 cm; volume 0.589 L) and their movement was not restricted.

The cockroaches were divided into three control (CON) and three experimental (EMF) groups according to the duration of EMF exposure: (1) 24 h EMF exposure; (2) 72 h EMF exposure and (3) 7 day EMF exposure. In each animal, escape reaction time was measured only once. Control groups of insects were handled in an identical manner (glass chamber was located in the shame exposure system for the same duration) to obtain similar experimental conditions, except for the presence of EMF. The temperature during experiments was monitored using thermocouples mounted under each exposure system.

### Heat plate apparatus and experimental procedure

**Fig. 2:**
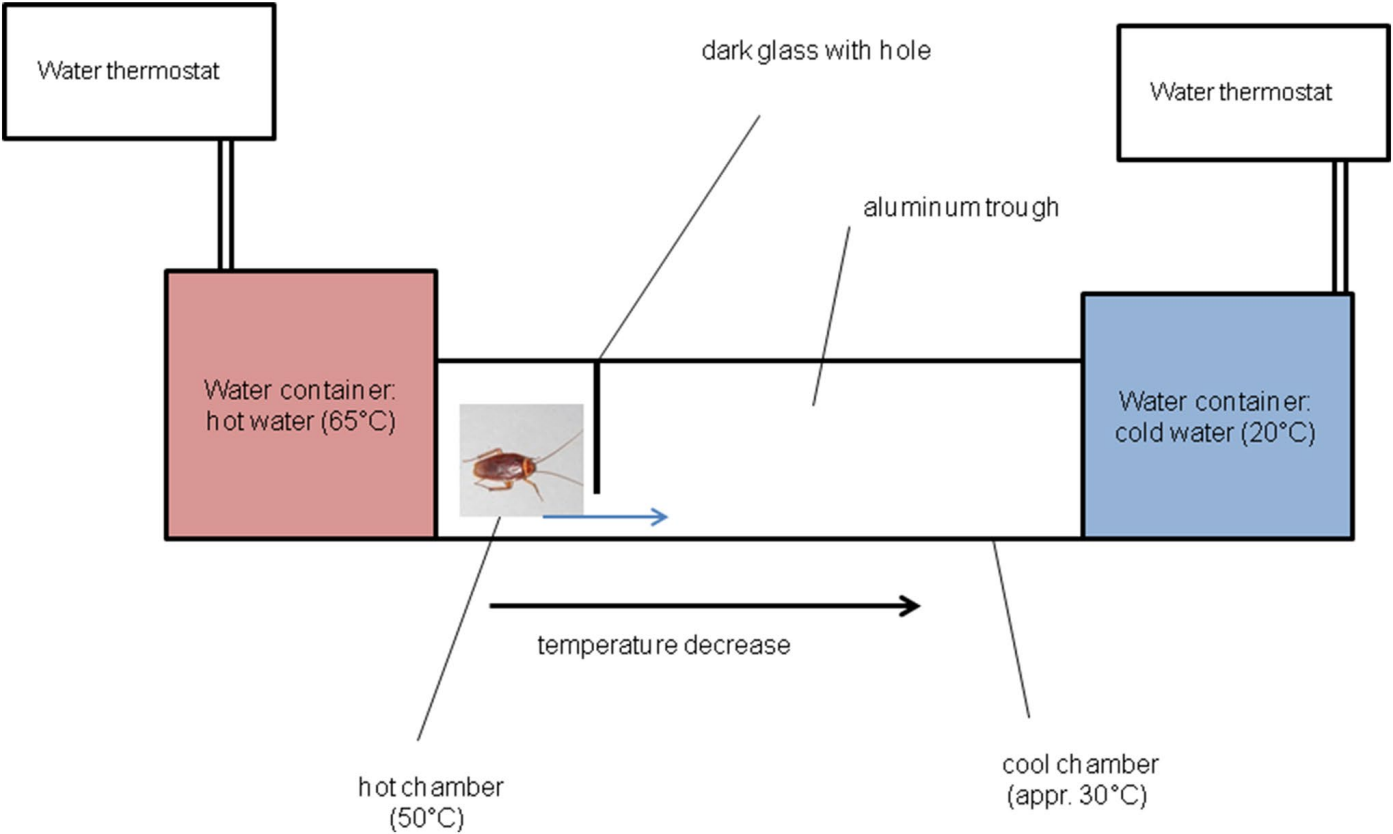
Scheme of the equipment used in the heat nociception assay

The heat plate apparatus consisted of two aluminum chambers: ‘hot’ one (50 °C) and ‘cool’ one (30 °C) (Fig. 2). Hot chamber adhered to the aluminum container filled with hot water (65 °C) pumped from water thermostat. This allowed to maintain a 50 °C inside the chamber. On the other end, there was a second aluminum container which was filled with cold water (20 °C) pumped from another water thermostat. The temperature decreased linearly from hot to cold end, and in the cool area a temperature of approximately 30 °C was maintained. Chambers were separated by 5-mm thick dark glass with a 1-cm hole that enabled the insect to escape. After placing the testing cockroach inside hot chamber, a dark glass top cover was located and the escape reaction time started to be measured. The end of escape reaction time was determined when the cockroach’s head appeared in the cold chamber.

### Sample preparation

Homogenates of the whole-body cockroaches were prepared using a glass Potter homogenizer (Kleinfeld Labortechnik, Gehrden, Germany). The samples were homogenized in ice-cold phosphate buffer pH 7.2 (Sigma) for 2–3 min and then were centrifuged at 12,000*g* for 10 min at 4 °C. Supernatants were used for determination of MDA content and reduced glutathione (GSH) concentrations.

### Malondialdehyde (MDA) assay

To determine lipid peroxidation level, the thiobarbituric acid reacting substance (TBARS) was measured according to the method of Buege and Aust (1978), modified by Cheeseman and Slater (1993) and expressed in terms of MDA content. The samples were incubated with 15% trichloroacetic acid (TCA) and 0.37% thiobarbituric acid (TBA). The mixture was heated on boiling water bath for 20 min with butylated hydroxytoluene (BHT) in ethanol that prevented from artefactual lipid peroxidation during the boiling step. After centrifugation (12,000×*g* for 15 min), the absorbance of samples was measured spectrophotometrically at 535 nm. The molar extinction coefficient used to calculate MDA concentrations was 156 mM^−1^ L^−1^ cm^−1^. MDA content was expressed as µM/mg tissue.

### Reduced glutathione (GSH) assay

To determine the reduced GSH concentration, the Ellman method (1959) was used. Whole-body homogenates were mixed thoroughly with a stock solution containing: 10% (TCA) and 10 mM ethylenediaminetetraacetic acid (EDTA) and were centrifuged for 10 min at 10,000*g*. After centrifugation, the supernatants were added to 2.3 mL of deionised water, 100 mL of 0.3M EDTA, 300 mL of 0.32M tris(hydroxymethyl)aminomethane (TRIS) and 100 mL of 0.086 mM 5,5′-dithiobis-2-nitrobenzoic acid (DTNB), and were maintained at 10 °C for 10 min. The absorbance of samples was measured spectrophotometrically at 412 nm. The GSH concentration was expressed in µmol/g tissue.

### Data analysis

All data were tested for normality (Kolmogorov–Smirnov test) and homogeneity of variance (Levene’s test). Escape reaction time was analyzed using Kruskal–Wallis test and pairwise comparisons were determined using Mann–Whitney *U* test. To assess the effect of EMF on lipid peroxidation and glutathione levels, two-way ANOVA was used with: (1) exposure to EMF and (2) duration of exposure as fixed factors, followed by pairwise comparisons with Bonferroni correction. In all cases, *p* < 0.05 was considered as statistically significant. All analyses were made using IBM SPSS Statistics 24 software.

## Results

### Electromagnetic field alters cockroaches’ response to noxious heat

**Fig. 3:**
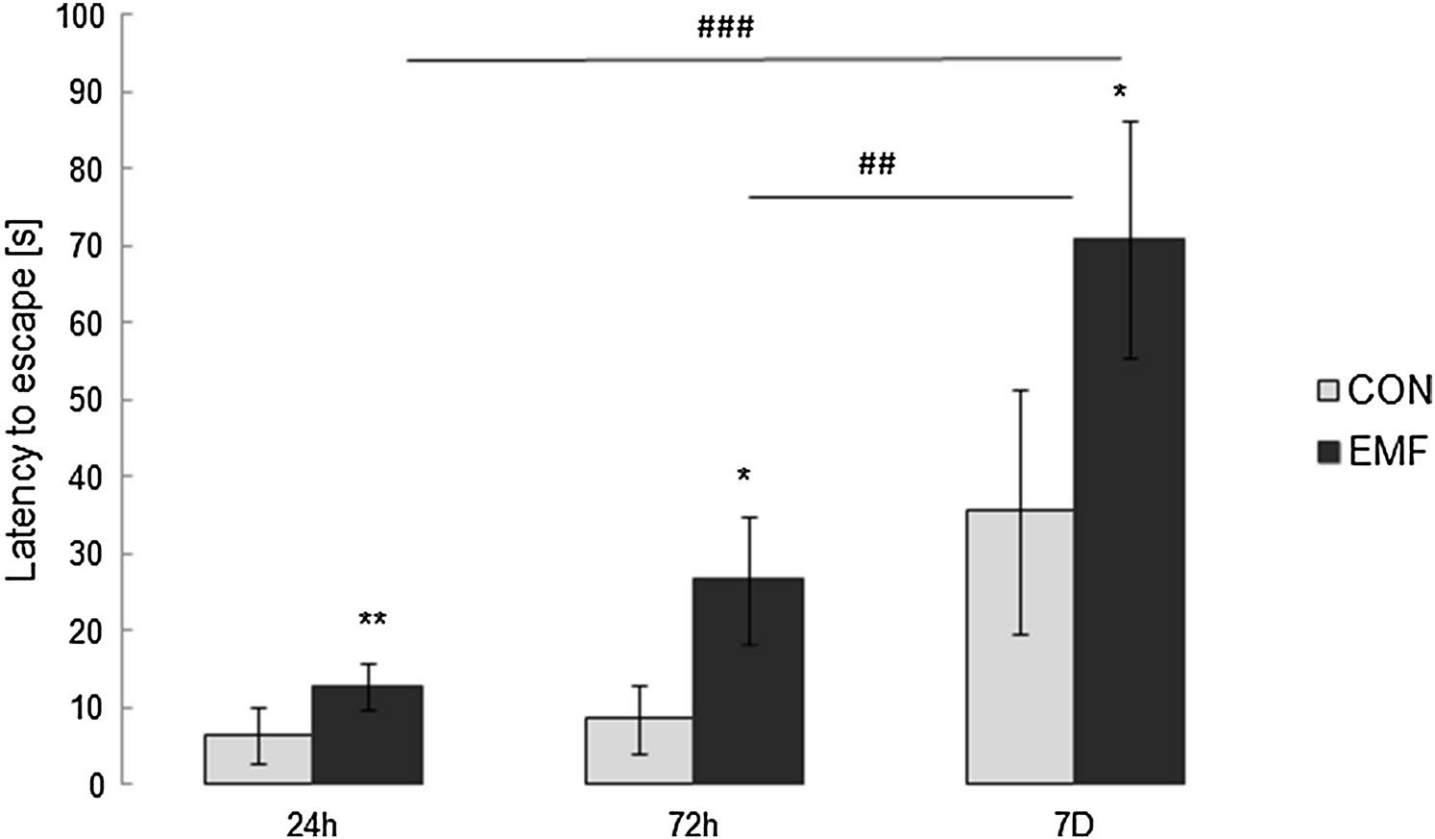
Latency to escape (s; mean ± SEM) from noxious heat in cockroach *Periplaneta americana* L. exposed to electromagnetic field (EMF) for 24, 72 h or 7 days. *Significant differences between EMF-exposed and control groups (*p < 0.05; **p < 0.01 vs. control group; Mann–Whitney *U* test; *n* = 20); ^#^significant differences between EMF-exposed groups (^##^*p* < 0.01; ^###^*p* < 0.001)

As shown on Fig. 3, exposure to electromagnetic field significantly affects the insect response to noxious high ambient temperature. Time of exposure to EMF (24 vs. 72 h vs. 7 days) revealed a significant effect on insects response to high ambient temperature (Kruskal–Wallis test: *χ*^2^ = 14.73; *df* = 2; *p* = 0.001). In control groups, significant increase in latency to escape from noxious heat with duration of exposure was also observed (Kruskal–Wallis test: *χ*^2^ = 15.04; *df* = 2; *p* = 0.001). However, in EMF-exposed cockroaches, significant prolongation of time spent at 50 °C comparing to control groups was observed. Escape reaction time in cockroaches exposed to EMF for 24 h was twice as long as observed in control insects (12.9 ± 3.0 s; Mann–Whitney *U* test: *U* = 68.0, *z* = − 3.43, *p* = 0.001). Time spent at noxious heat in cockroaches exposed to EMF for 72 h tripled in comparison to value for control group (26.8 ± 8.2 s; Mann–Whitney *U* test: *U* = 92.5, *z* = − 2.19, *p* = 0.03). Increase in latency to escape was also observed in cockroaches exposed to EMF for 7 days (70.8 ± 15.4 s; Mann–Whitney *U* test: U = 14.5, *z* = − 2.23, *p* = 0.02 comparing to control group).

### Exposure to EMF induces oxidative stress

**Fig. 4:**
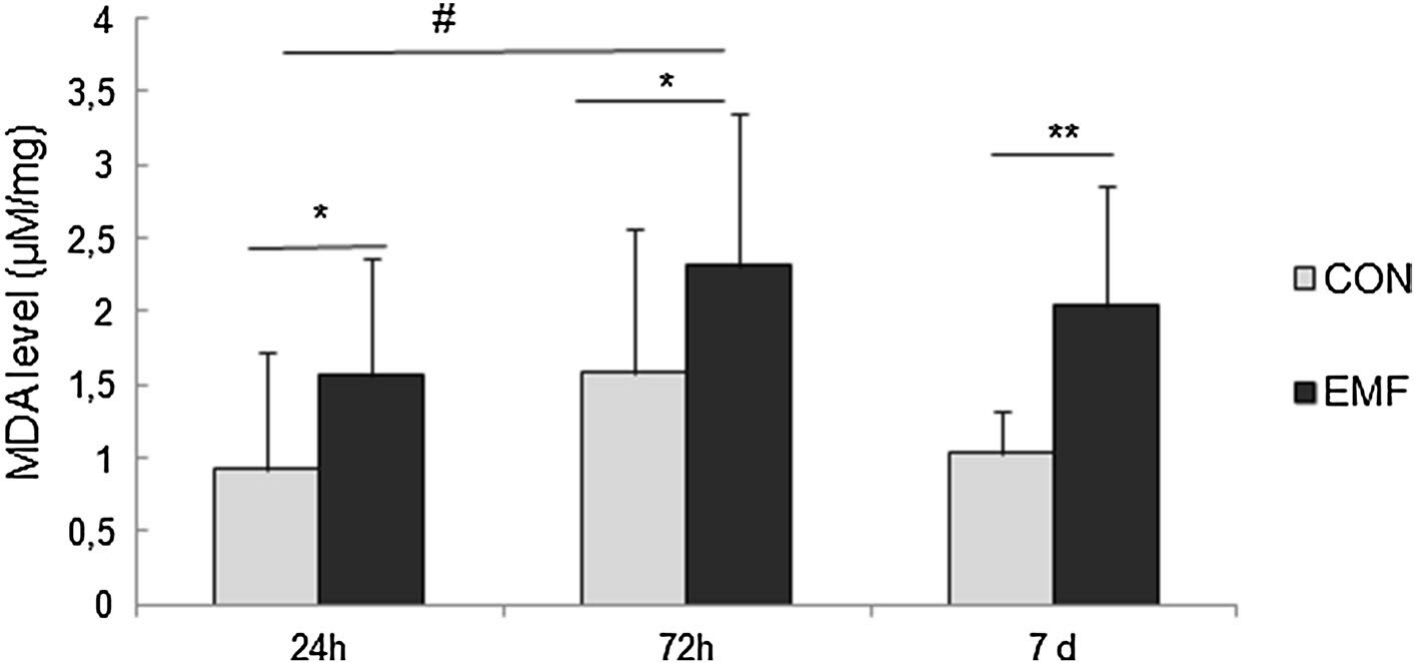
MDA levels (µmol/g; mean ± SD) in cockroaches exposed to electromagnetic field (EMF) for 24, 72 h or 7 days. *Significant differences between EMF-exposed and control groups (*p < 0.05, **p < 0.01); # indicates significant differences between EMF-exposed groups (^#^*p* < 0.05)

MDA levels in cockroaches were significantly increased after exposure to EMF and their value depended on the time of exposure (Fig. 4). Two-way ANOVA showed that EMF exposure affects MDA level (*F*_1,91_ = 17.59, *p* < 0.001). However, there was no significant interaction between exposure to EMF and its duration (*F*_2,97_ = 0.28, *p* = 0.75). Significantly elevated MDA levels in comparison to control group were observed after 24 h (1.56 µM/mg; *p* = 0.02), 72 h (2.31 µM/mg; *p* = 0.03) and 7 days of exposure (2.04 µM/mg; *p* = 0.003). The highest MDA level was observed in cockroaches exposed to EMF for 72 h and was significantly higher than that observed in cockroaches after 24 h of EMF exposure (*p* = 0.04).

### Exposure to EMF reduces glutathione levels

**Fig. 5:**
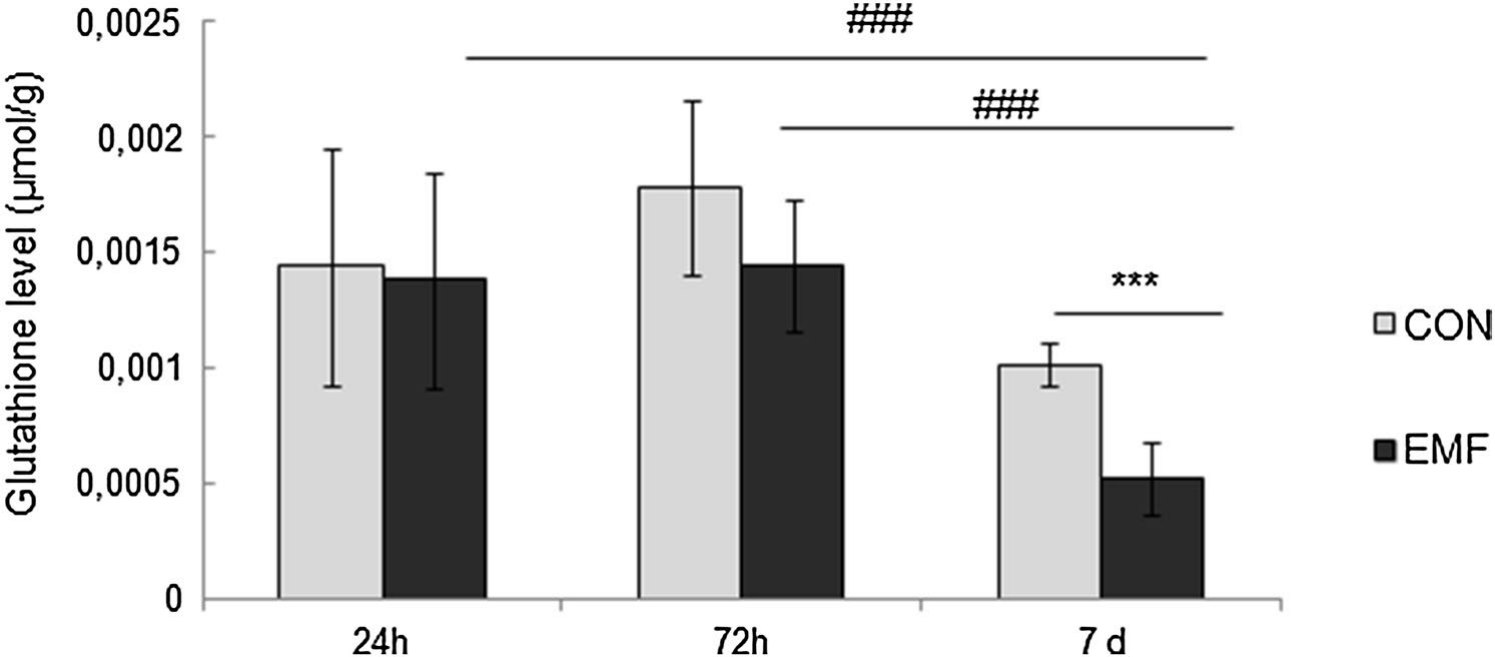
Glutathione levels (µmol/g; mean ± SD) in cockroaches exposed to electromagnetic field (EMF) for 24, 72 h or 7 days. *Significant differences between EMF-exposed and control groups (***p < 0.001); # indicates significant differences between EMF-exposed groups (^###^*p* < 0.001)

Exposure to electromagnetic field resulted in significant decrease in glutathione levels in the examined cockroaches (two-way ANOVA: *F*_1,72_ = 5.97, *p* < 0.05). 24- and 72-h exposure to EMF did not affect the glutathione levels compared to control groups (Fig. 5). Marked effect of EMF was observed after 7 day exposure, observed as decline in glutathione level comparing to control cockroaches (*p* < 0.001). Significant difference in glutathione level was also observed between cockroaches exposed to EMF of different durations. The lowest value of GSH was observed after 7 days of exposure and it was significantly different from that observed after 24 h exposure (*p* < 0.001) and 72 h exposure (*p* < 0.001).

## Discussion

The results of our study demonstrate that in cockroaches exposed to electromagnetic field, the response to noxious heat is altered. The longer the exposure to EMF was continued, the stronger effect was observed. After exposure to stressful stimuli, the phenomenon of pain suppression is observed, known as stress-induced analgesia (Butler and Finn 2009). Exposure to EMF affects both pain sensitivity and pain inhibition. Increased pain sensitivity after exposure to a different ranges of magnetic environments has been shown to occur in a variety of animal species, including humans (Jeong et al. 2000). EMF exposure has been shown to reduce both exogenous, as well as endogenous opioids effects in mediating analgesia. However, the effect of EMF on nociception depends on its intensity and duration of exposure (Del Seppia et al. 2007). For example, in the land snail *Cepaea nemoralis*, the EMF may reduce, but have no effect or induce opioid-mediated analgesia (Prato et al. 2000).

Stress in insects including thermal stress leads to a marked increase of oxidative stress as well as of heat shock protein (HSP) levels (Barclay and Robertson 2000; Robertson 2010) that play a key role in thermoprotection.

Numerous studies have shown that exposure to EMF increases oxidative stress in mammals (Consales et al. 2012). Our results clearly show that oxidative stress is a response to EMF exposure also in American cockroach. EMF induced the increase of MDA level, a marker of lipid peroxidation, in cockroach. The increase in this lipid peroxidation marker was observed after 24-h exposure and remained elevated until 7-day exposure. Zhang et al. (2016) have shown that effect of EMF on MDA levels is sex-dependent. They observed decline in MDA level in male, but not in female *Drosophila* exposed to EMF (50 Hz, 3 mT) for 12 h. However, in our experiments, the intensity and duration of EMF exposure was higher, what could act as a marked stressor.

In our studies, we evaluated the level of low molecular antioxidant glutathione. The short-term EMF exposure did not affect its level. However, the prolonged exposure resulted in the glutathione decline. Reduced glutathione levels after EMF exposure was observed in mice (Arendash et al. 2010) and guinea pigs (Meral et al. 2007). Our results demonstrate that EMF (50 Hz, 7 mT) exposure may act as a stressor inducing oxidative stress observed as increase of the lipid peroxidation level and reduction of the glutathione level. However, how the EMF-induced changes in oxidative state are related to the function of nociceptors need to be further elucidated.

There are also reports showing that EMF affects heat shock protein (HSP) accumulation in cells (Tokalov and Gutzeit 2004; Alfieri et al. 2006; Bernardini et al. 2007; Li et al. 2013; Wyszkowska et al. 2016). Thus, the increase in latency to escape from heat in the examined cockroaches may be a result of heat shock proteins accumulation, which act as molecular chaperones and help denaturated proteins to refold. It was suggested that a cellular response to EMF mimics the heat shock response (Kang et al. 1998); however, the data are inconsistent. Extremely low frequency magnetic fields affect heat shock proteins (HSPs) accumulation in cells, what suggests that at the molecular level, stress processes are affected by exposure to high levels of EMF (50 Hz, 680 µT–7 mT) (Alfieri et al. 2006; Wyszkowska et al. 2016). Recent studies on the effect of EMF of over 1 mT intensity have shown an increase in HSP70 transcription that affected neuronal activity in mice (Sun et al. 2016). On the other hand, Morehouse and Owen (2000) showed no significant effect of EMF (60 Hz, 8µT) on HSP70 level in HL60 cells. The studies on chick embryos have shown that repeated exposure to EMF (60 Hz, 8µT) led to reduced HSP70 levels and decline in cryoprotection (Carlo et al. 2001). These data suggest that the effect of EMF on HSP level depends on the type of the cell and dose of EMF (frequency and density, as well as duration exposure).

In summary, our results proved that EMF alters the response of cockroaches to noxious heat. We presume that research on cockroach model in determining the role of EMF in pain sensitivity would be a useful tool for developing the strategies for pain inhibition.

## Abbreviations

EMF: Electromagnetic field
GSH: Glutathione
HSP: Heat shock protein
MDA: Malondialdehyde
TRP: Transient receptor potential
